# Evaluating a knowledge translation tool for parents about pediatric acute gastroenteritis: a pilot randomized trial

**DOI:** 10.1186/s40814-018-0318-0

**Published:** 2018-08-02

**Authors:** Lauren Albrecht, Shannon D. Scott, Lisa Hartling

**Affiliations:** 1 Department of Pediatrics, University of Alberta, Edmonton Clinic Health Academy, 11405 87 Avenue, Edmonton, Alberta T6G 1C9 Canada; 2 Faculty of Nursing, University of Alberta, 3rd floor Edmonton Clinic Health Academy, 11405 87 Avenue, Edmonton, Alberta T6G 1C9 Canada

**Keywords:** Pilot trial, Pediatric emergency care, Health consumers, Parents, Caregivers, Knowledge translation

## Abstract

**Background:**

Pediatric acute gastroenteritis (AGE) is a common childhood illness with substantial health, family, and system impacts. Connecting parents to evidence-based patient education is key to effective decision-making and therapeutic management of AGE. Digital knowledge translation (KT) tools offer a promising approach to communicate complex health information to parents; therefore, we developed a whiteboard animation video for parents about AGE. To optimize future effectiveness evaluation of this video, the purpose of this pilot study is to assess feasibility of effectiveness outcomes and specific trial methods in four key trial domains.

**Methods:**

A single-site, parallel-arm, pilot randomized trial will be conducted. The trial will employ quantitative and qualitative methods to evaluate feasibility objectives in key scientific, process, management, and resource domains. Parents seeking care for a child with AGE in the emergency department (ED) over a 6-month period will be randomized to receive the whiteboard animation video or a sham control video. Quantitative data will be collected electronically in the ED and at home (4–10 days post-ED visit). Qualitative data will be collected via semi-structured interviews with experimental condition participants after quantitative data collection. Data will be collected to perform a sample size calculation for a full-scale trial. Scientific outcomes will include parental knowledge, decision regret, and health utilization, and estimation for these outcomes will use confidence intervals (CI) of different widths to illustrate strength of preliminary evidence. CIs will be presented alongside minimum clinically important differences (MCIDs) calculated using two methods: (1) data driven and (2) patient perspective. Descriptive statistics will be calculated to describe process, management, and resource domain outcomes. Qualitative thematic analysis will be conducted to describe additional process, management, and resource outcomes in the experimental group. Analyses will be performed using intention-to-treat.

**Discussion:**

This pilot randomized trial will inform the design and conduct of a full-scale, effectiveness trial by gathering key data in four domains: scientific, process, management, and resource. These results will impact the emerging field of KT efforts targeting health consumers and advance the science on the best mode of patient education for acute childhood illnesses.

**Trial registration:**

clinicaltrails.gov registration number NCT03234777. Registered 31 July 2017.

**Electronic supplementary material:**

The online version of this article (10.1186/s40814-018-0318-0) contains supplementary material, which is available to authorized users.

## Background

Pediatric acute gastroenteritis (AGE), characterized by vomiting and diarrhea, is a common presentation in emergency departments (EDs) and remains a leading cause of global pediatric morbidity [1–4]. In Canada and other developed countries, AGE is most often caused by viruses [1]. Annually, there are five million cases of pediatric AGE in Canada and it represents 10% of Canadian pediatric ED visits, resulting in a yearly healthcare cost of $3.7 billion [5]. In addition to substantial health system impacts, pediatric AGE affects families in a multitude of ways, including negative effects on physical and emotional wellbeing of children and parents [6] and frequent parental work loss [7].

A recent Canadian, qualitative study of parents/caregivers (*n* = 15) highlighted the “real-life” complexity that influences health decision-making for pediatric AGE (i.e., past experiences and life circumstances) as well as AGE-related information needs, which included symptom management, understanding the normal course of illness, the cause of illness, information specific to dehydration, where to purchase helpful items, and how to talk to their child about AGE [8]. An American, cross-sectional study that evaluated parent/caregiver (*n* = 229) knowledge about AGE indicated a wide variation in knowledge levels and demonstrated that knowledge was positively correlated to accessibility of health information, level of education, ethnicity, and experience with dehydration. This study recommended that future education interventions should be designed to improve general knowledge [9]. A Canadian non-randomized trial targeting parents/caregivers (*n* = 105) of children with AGE in the ED evaluated one-on-one nursing education session in the ED and an educational home visit versus no intervention control. The study found a small (not statistically significant) increase in knowledge at 1 month, but this change was not sustained at 6 months [10]. A French cluster randomized trial targeting adults and parents/caregivers (*n* = 400) with children with either tonsillitis or AGE evaluated the effect of patient information sheets. This study found statistically significant, positive effects on behavior (primary) and knowledge (secondary) outcomes in the information sheet child sub-group compared to the no information, control child sub-group 10–15 days post-intervention [11].

Knowledge translation (KT) is defined as the synthesis, exchange, and application of knowledge to improve the health of individuals, provide more effective health services and products, and strengthen health care systems [12]. Current approaches to KT research are largely focused on aligning the behaviors of health professionals with best research evidence; however, there are increasing calls for KT interventions for health consumers to influence health knowledge, decision-making, and service utilization [13], especially given the many alternative health options open to parents through the Internet. At present, there is little guidance on the most effective approach, content, duration, and intensity of education for this diverse population [14–16]; however, it is hypothesized that providing evidence-based child health information to parents and families has the power to ensure consistent parental management of child health over time and across settings [17], increase effective health decision-making [18], and reduce health system costs [18]. It is critical to generate empiric evidence to help determine the optimal modes for delivering evidence-based information to parents and families.

At present, it is routine practice to provide health education to parents seeking care for their children in EDs to guide care after discharge [17]. Typically, education is provided at the end of the emergency department visit when parents are tired and anxious to leave, making them less likely to ask important questions and retain information [19]. Additionally, information provided verbally is often brief [20] and written information is often too complex for most adults to comprehend [21, 22].

Since online tools (e.g., podcasts, e-books, animations, and infographics) are low or no cost, easily accessible, and can be consumed on demand, they hold promise as an effective approach to KT for parents and caregivers seeking research-based information related to child health [23, 24]. Additionally, online platforms allow content to be viewed as frequently as needed, which may improve information retention and compliance [24].

To address the research-practice gap in pediatric AGE and provide parents with a reliable, research-based information resource, our research team developed a 3-min whiteboard animation video using patient-oriented research methods [25]. Whiteboard animation videos are short, hand-drawn, and narrated videos optimized for online streaming. Video content was drawn from knowledge synthesis results of treatment and management strategies for pediatric AGE [1]. The video storyline was developed from a qualitative study (*n* = 15) that gathered and synthesized stories from parents/caregivers of children with vomiting and diarrhea who visited an ED for healthcare [8]. Video prototypes were reviewed, and a final version approved by pediatric emergency clinicians, research nurses, and parents. The video depicts a family with a sick child trying to determine whether medical attention is required. The family recalls an information sheet given to them at a hospital when their child was ill with the same symptoms at an earlier time. The family assesses their child using the information sheet and determines to monitor and manage the child’s illness at home until symptoms resolve. The family describes worsening symptoms that would require them to take their child to the emergency department. Artists, including a script writer, animators, and voice actor, were contracted to work with the research team to ensure high-quality video production.

Rigorous evaluation of the effectiveness of the whiteboard animation video is a critical next step. Given some uncertainty regarding the most appropriate methods for evaluating the effectiveness of KT tools for parents [26], including best outcome measures and parameters for sample size calculations, we have chosen to first undertake a pilot trial [27–29]. We are also interested in exploring the feasibility of using an electronic platform to assist with recruitment, intervention delivery, and data collection. The study objectives are listed in Table 1 according to four key pilot trial domains [27].

**Table 1:** Study objectives

Pilot trial domains	Study objectives
Scientific domain	• To estimate the potential effectiveness of a digital knowledge translation tool for parents/caregivers about pediatric AGE. Effectiveness outcomes include knowledge, decision regret, and post-ED healthcare utilization.
	• To identify the perceived benefit and value of KT tools for this population, including important components that enhance knowledge about childhood AGE and decision-making regarding choice to take child with AGE to the ED.
Process domain	• To identify whether using an electronic, web-based platform for intervention delivery and data collection is feasible with this population.
Management domain	• To describe this population’s willingness to participate in future, similar research (i.e., full-scale trial).
Resource domain	• To estimate time required for participants to complete data collection forms.
	• To identify whether using iPads to collect data is feasible with this population.

## Methods

This is a parallel-arm, randomized, pilot trial. Convenience sampling will be used to recruit parents/caregivers seeking care for a child with vomiting and diarrhea in the ED and randomize them to receive the intervention of interest (i.e., whiteboard animation video) or a sham control condition (i.e., standard video of similar length). Data will be collected using quantitative and qualitative methods over a 6-month period. This protocol has been registered on clinicaltrails.gov (NCT03234777) and has obtained ethics approval from the University of Alberta Health Research Ethics Board and operational approval from the provincial health authority.

### Study location and population

The study will be conducted at one Canadian, tertiary care, pediatric hospital. Parents meeting the eligibility criteria in Table 2 will be invited to participate.

**Table 2:** Study eligibility criteria

Study eligibility criteria	
Inclusion criteria	1. Parent or caregiver of a child 16 years old or younger.
	2. Child is presenting to the ED with vomiting and diarrhea.
	3. Parent is fluent in English.
	4. Parent is willing to be contacted for follow-up data collection.
Exclusion criteria	1. Child has significant chronic gastrointestinal problem or inflammatory bowel disease (i.e., Crohn’s disease, inflammatory bowel disease, ulcerative colitis, chronic constipation).
	2. Child is taking immunosuppressive therapy or known history of immunodeficiency.
	3. Child has undergone oral or gastrointestinal surgery within the preceding 7 days.
	4. Child has had a prior visit to the ED for vomiting and diarrhea within the preceding 14 days.

### Recruitment

**Fig. 1:**
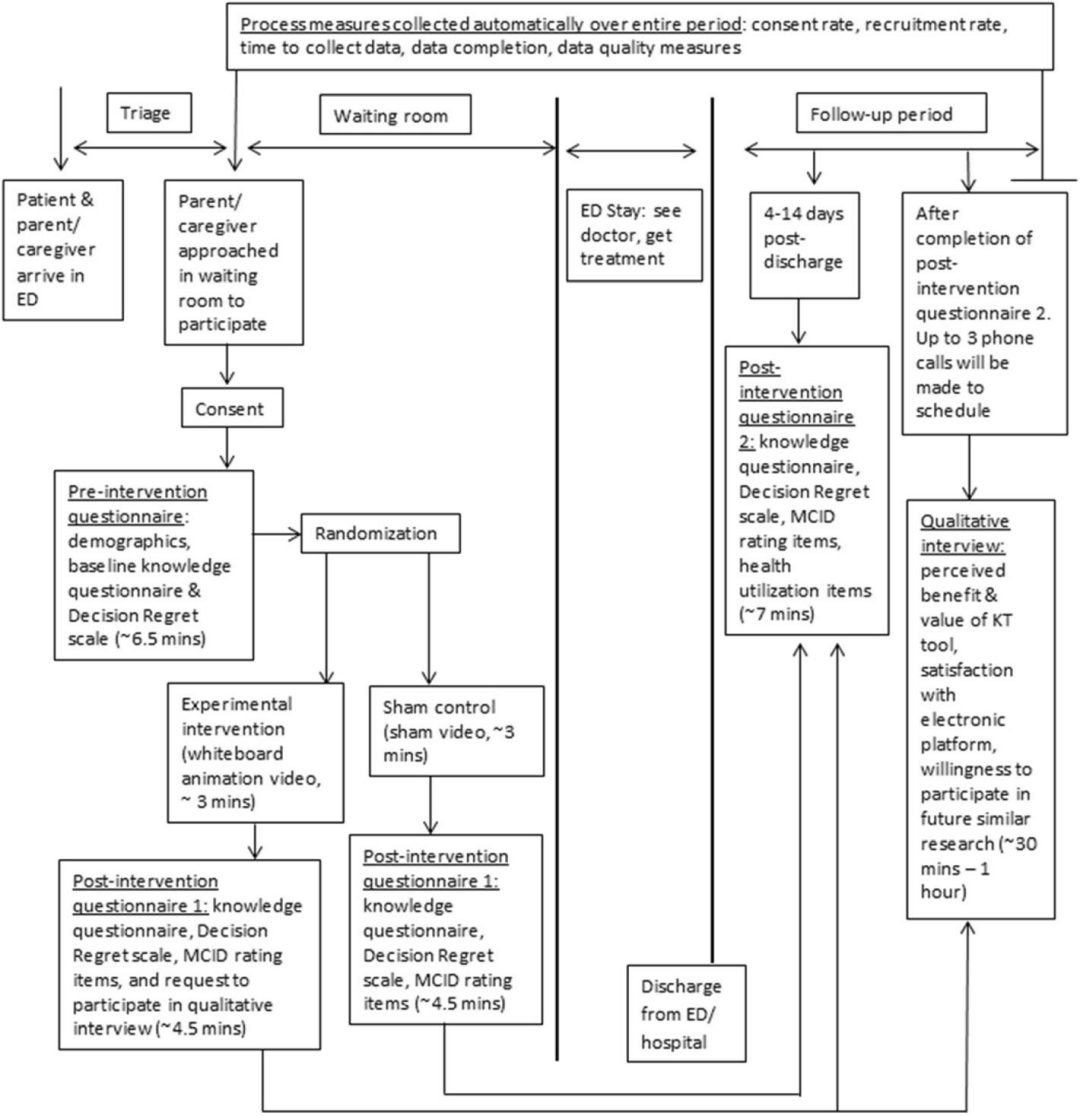
Trial flow and timing of data collection

ED triage records will be screened in real time to identify potential study participants. The parent/caregiver of consecutive individuals with primary complaint of vomiting and diarrhea will be approached in the ED waiting room post-triage assessment (Fig. 1) during data collection recruitment hours (0900–2300 Monday to Friday, 1500–2300 Saturday and Sunday). A member of the study team will assess inclusion/exclusion criteria and review the study information letter. Informed consent will be indicated on iPads as part of the electronic data collection platform.

### Interventions

The interventions take place in the ED waiting room post-triage assessment and prior to consultation with a physician. Post-intervention, all participants will receive standard medical care from healthcare providers. The study hospital has an information sheet about AGE; however, education about AGE including whether the information sheet is given to parents/patients is at the discretion of the attending physician.

For this study, the experimental intervention is a 3-min, whiteboard animation video about a family’s experience with pediatric AGE (URL: https://www.youtube.com/watch?v=5N3gMtvkCqk&list=PLxWz0fEGuv6o5LrEpvdsmQFs5zB1dIFR8&t=17s&index=12). The sham control condition is a 3-min standard video developed by the Centres for Disease Control and Prevention about hand washing for infection control (URL: https://www.cdc.gov/cdctv/healthyliving/hygiene/fight-germs-wash-hands.html) [30]*.* Parents will view either the experimental or sham control intervention once on an iPad in the ED waiting room post-triage assessment. Disposable headphones will be provided with the iPads to maintain blinding. At the end of data collection in the ED (post-intervention questionnaire 1), parents can opt to receive a link to their assigned video via email to view as often as they wish from their own device(s). Parents from the experimental intervention group that are participating in the qualitative interview will view the whiteboard animation video again at the start of the interview.

### Randomization

Blocked randomization with randomly chosen block sizes will be used to ensure equal distribution of participants to the experimental and sham intervention study arms [31]. The blocked randomization sequence will be computer generated. Following sequence generation, the randomization sequence will be entered into a confidential module on the electronic platform. Once the sequence is entered, the randomization module will only be accessible using a confidential password. The randomization sequence will be kept confidential. It will be inaccessible to data collectors/outcome assessors and to the study participants.

After completing the pre-intervention questionnaire, including demographic information and baseline outcome data, on the electronic data collection platform, individual parents/caregivers will automatically be randomized to one of the study conditions (i.e., experimental intervention or sham control) based on the randomization sequence. This process will be seamless to participants. After viewing the study condition materials, participants will be automatically directed to post-intervention questionnaire 1. At study completion, all participants will receive a thank you email with links to both videos shown in the study.

### Blinding

Using an electronic platform for data collection, study group allocation, and intervention viewing will allow participants to access the interventions and provide data independent of the research team. Participants and study staff will be blind to how the content on the iPads differs between groups during data collection.

### Outcomes

A detailed description of study outcomes and outcome measures is presented in Table 3. KT tools are intended to impact end-user experience, including increasing knowledge, influencing healthcare decision-making, and use of healthcare resources/services. Comprehension and retention of health information is a key component of the patient experience, a determinant of care instruction follow-through, and the cornerstone of health literacy; therefore, parental knowledge of childhood AGE (e.g., cause of AGE, signs of dehydration, management of dehydration) over time will be assessed. In addition to comprehension, educational materials may also influence health decision-making; therefore, parental decision regret over time will be assessed to examine this impact considering the decision to bring their child to the ED for care. Additionally, minimum clinically important differences (MCIDs) will be identified for both knowledge and decision regret outcome measures. Finally, healthcare utilization post-ED visit will be explored as a potential future outcome.

**Table 3:** Study outcomes, outcome measures, and data analysis methods by domain

Study domain	Outcomes and outcome measures	Data analysis methods
Scientific domain	*Quantitative*: Parental knowledge will be evaluated using an 8-item knowledge questionnaire developed by the research team. This questionnaire was informed by the abridged Caregiver Gastroenteritis Knowledge Questionnaire (CGKQ) [55] and tailored to key content in the experimental intervention video. It has been piloted with the target population (*n* = 15) and revised for clarity. It takes approximately 2.5 min to complete.	Correct responses will be given a score of one and are summed for a final score (0–8).
	*Quantitative*: Decision regret will be measured by the Decision Regret Scale comprised of five, 5-point Likert items. Internal consistency and validity have been demonstrated and it is a useful tool for measuring regret after health care treatment decisions, is easy to administer, takes less than 1 min to complete, and results in very few missing responses [56].	Items are scored individually and converted to a 0–100 scale following instructions in the user manual [57]. Items are summed and averaged for a final score, with regret increasing with a higher score.
	*Quantitative*: MCIDs will be calculated for the parental knowledge questionnaire and the decision regret scale using two methods: (1) data-driven approach and (2) patient-perspective [58].	Using a data driven approach, the standard error of measurement will be used as a proxy for the MCID. Using a patient perspective, patients will make global ratings on their own change via Likert items in post-intervention questionnaires 1 and 2. Both approaches will be compared to quantified changes in outcome measures and absolute change for both will be calculated [58].
	*Quantitative*: Post-ED healthcare utilization will be assessed by three items developed by the research team.	Descriptive statistics will be calculated.
	*Quantitative*: The perceived benefit and value of the KT intervention for pediatric AGE will be evaluated using four items developed by the research team and by examining the number of participants that request a video link after questionnaire 1.	Descriptive statistics will be calculated.
	*Qualitative*: The perceived benefit and value of the KT intervention for pediatric AGE will be also evaluated via a qualitative interview with a convenience sample of volunteer parents from the experimental intervention group.	Thematic analysis of interview data will be conducted.
Process domain	*Quantitative*: Consent rate will be measured by (1) percentage of people approached who consent to participate and (2) timing (date, time of day) of refusals.	Descriptive statistics will be calculated.
	*Quantitative*: Recruitment rate will be measured by (1) percentage of people who consented to participate and complete pre-intervention questionnaire, post-intervention questionnaire 1, post intervention questionnaire 2, and qualitative interview; and (2) timing (date, time of day) of recruitment.	Descriptive statistics will be calculated.
	*Quantitative*: Data completion will be measured by (1) percentage of missing/blank survey items and (2) percentage of drop-outs at post-intervention questionnaire 2.	Descriptive statistics will be calculated.
	*Qualitative*: Satisfaction with electronic platform will be evaluated in the qualitative semi-structured interview with a convenience sample of volunteer parents from the experimental intervention group.	Thematic analysis of interview data will be conducted.
Management domain	*Quantitative*: Data quality will be measured by number, type, and duration of technical problems (i.e., error messages, problems with internet connectivity, and lost data) with an online platform throughout the study period.	Descriptive statistics will be calculated.
	*Qualitative*: Parents’ willingness to participate in future, similar research will be evaluated by a qualitative interview with a convenience sample of volunteer parents from the experimental intervention group.	Thematic analysis of interview data will be conducted.
Resource domain	*Quantitative*: Time to collect data will be measured by (1) average length of time to complete study questionnaires and (2) average length of time (days) to complete post-intervention questionnaire 2 post-discharge.	Descriptive statistics will be calculated.
	*Quantitative*: The feasibility of using iPads to collect data in the ED with parents/caregivers will be measured by tracking the number of broken, lost, or stolen iPads, iPad chargers (including cord and plug), iPad cases, and WiFi hubs.	Descriptive statistics will be calculated.

### Sample size estimate

Sample size calculations are not required for pilot/feasibility studies as hypothesis testing is not the focus of this research design [32, 33]. Rather, recruitment will take place over a 6-month period and will be evaluated as part of the identified process outcome measures. This 6-month period is intended to reflect the seasonal nature of viral gastroenteritis, the most common cause of infection, in temperate climates [34]. Recruitment will take place over the peak infection time of late winter [34]. The study site has approximately 500 patients presenting with symptoms of AGE over a 6-month period.

Philosophically, qualitative methods do not conduct prospective determinations of a sample size; instead, an adequate sample permits a deep, case-oriented analysis that results in a new understanding of experience [35]. In this study, convenience sampling will be used to recruit participants. It is anticipated that 12–20 interviews will be sufficient to see patterns in experiences [36].

### Data collection

Participants will be provided with disposable headphones and an iPad containing the iCare Adventure electronic platform. iCare Adventure is a client-server based e-therapeutics platform designed to expedite and improve the management of patients’ care within pediatric ED facilities. iPads, which are locked to the iCare Adventure app, are given to parents participating in research in the ED. All content within the app, including screen flow, textual content, images, videos, protocols, and questionnaires, is controlled on a centralized server. When the application restarts, it calls to the server, the iPads report all user interactions back to the server in real time, and the server can dynamically create real-time reports of the aggregated data. Data collection processes and forms will be piloted with the research team prior to the start of data collection.

The informed consent process and quantitative data collection will be completed on iCare Adventure, and participants will be automatically given the questionnaires and appropriate intervention (i.e., whiteboard video or sham video) based on the randomization sequence. A unique study identifier will be generated for each participant within the iCare Adventure platform. All data collection points will be electronically time-stamped.

As part of the post-intervention questionnaire 1, participants receiving the experimental intervention will be asked about their willingness to be contacted later for an individual, in-depth, semi-structured, qualitative interview. If interested, they will provide contact information (i.e., name, phone number, and best time to contact) and a member of the research team will follow up via telephone.

Using a qualitative descriptive approach [37, 38], a semi-structured interview guide will be used. This data collection strategy will be used to obtain all information required, probe participants’ responses, and give participants freedom to respond and illustrate concepts in an open-ended fashion [36]. Interview questions will move from the general to the specific, with interviews later in the data collection period becoming increasingly more focused [39]. Interviews will be conducted in-person or over the telephone and will be audio recorded. Audio recordings will be anonymized, de-identified, and transcribed verbatim by a third party contractor. Transcriptionists will sign a confidentiality agreement.

The following data will be collected over the course of this study:

1. Pre-intervention questionnaire (baseline, see Additional file 1): Participants will complete a pre-intervention questionnaire that includes demographics, knowledge questionnaire, and Decision Regret Scale within the iCare Adventure platform on the iPad.
  **Participants will then be randomized to view the study intervention or the standard care intervention within the iCare Adventure platform on the iPad. This process will be seamless for the participants.
2. Post-intervention questionnaire 1 (immediate, see Additional file 2): After viewing the intervention, participants will complete the knowledge questionnaire and Decision Regret Scale a second time. In addition, participants will complete two items assessing their own performance on the knowledge questionnaire and Decision Regret Scale and one item regarding the perceived value and benefit of the KT tool. They will also be asked if they would like a video link emailed to them. At the end of this questionnaire, parents will be informed that the post-intervention questionnaire 2 will be emailed to them 4 days after this ED visit for completion at their earliest convenience. Experimental intervention group parents will be asked about participation in a qualitative focus group at this time.
3. Post-intervention questionnaire 2 (4–14 days post-ED, see Additional file 3): Participants will be emailed a secure link on day 4 post-ED discharge to complete the knowledge questionnaire and Decision Regret Scale a third time, two items assessing their own performance on the knowledge questionnaire and Decision Regret Scale, three items related to healthcare utilization, and three items related to the perceived value and benefit of the KT tool (if applicable). Reminders to complete post-intervention questionnaire 2 will be sent to those who have not completed the survey every third day (day 7, 10, 13) to complete the survey by day 14 post ED-discharge. Previous research has demonstrated that 82% of AGE cases are resolved in 3 days or less and 14 days [40] represents the outer limit for pediatric AGE resolution [41].
4. Post-intervention semi-structured interview (sub-sample of experimental group, see Additional file 4): Participants in the experimental group indicating willingness to participate in an in-depth, semi-structured, qualitative interview will be contacted via telephone after completion of post-intervention questionnaire 2. Up to three phone calls will be made to establish interview date/time. Qualitative interviews will focus on satisfaction with iCare Adventure platform, perceived benefit and value of the KT intervention, and willingness to participate in future, similar research.

### Data analysis

All data will be aggregated and analyzed. Quantitative data will be downloaded from a secure Canadian server to SPSS for data cleaning and analysis [42]. Data cleaning measures may include recoding into categorical variables and comparing and recoding free-text responses where appropriate. Descriptive statistics and estimation are the recommended focus of pilot/feasibility trials [43, 44]. Descriptive statistics (e.g., frequencies and measures of variation and spread) will be calculated to describe the study groups. Analyses by outcome measure are presented in Table 3. Analyses will be conducted based on intention-to-treat.

Initial data will be collected to perform a sample size calculation for a full-scale trial [43]. Estimation for knowledge and decision regret outcomes will focus on calculating confidence intervals of different widths to illustrate strength of preliminary evidence [43, 44]. Confidence intervals for the difference of means (paired) will be presented for both potential effectiveness outcome measures to account for repeated measures [45]. The confidence interval for the difference of means from time 1 to time 2 for both groups will be calculated to examine initial change scores and the difference of means from time 2 to time 3 for both groups will be calculated to examine sustained change score. Confidence interval widths for both initial and sustained change measures will be set at 99, 95, 90, 85, 80, and 75% and presented together alongside a minimum clinically important difference (MCID) in a graph [46]. If each confidence interval crosses both 0 and the MCID, this is inconclusive evidence of effect; however, if each confidence interval both excludes 0 and crosses the MCID, there is evidence of a potentially clinically important difference. A confidence interval that is above or equal to the MCID indicates that at this level there is a clinically meaningful difference between the groups.

Qualitative data will be de-identified during verbatim transcription. Prior to analysis, transcripts will be checked with the audio files for accuracy. Qualitative data will be managed and analyzed using NVivo data management software [47]. Qualitative outcomes will be analyzed using thematic analysis by breaking interview text into small units for a detailed, nuanced account of the data [48–50]. This iterative process will be concurrent to data collection [37]. Thematic analysis will be guided by the hybrid approach of inductive and deductive coding and theme development described by Fereday and Muir-Cochrane [51]. Deductive coding of the interview transcripts will be done first using the semi-structured interview guide as a framework; smaller units of data that emerge inductively will be coded for increased granularity and specificity. To ensure analytic rigor, field notes will be collected during the data collection and analysis process and coded alongside interview data [48, 52, 53].

### Data storage and security

All data will be stored on secure Canadian servers that are compliant with data privacy and security regulations to safeguard medical information as per the Health Insurance Portability and Accountability Act of 1996 [54]. The server is protected by a firewall and is backed up daily. All data stored in the server database is anonymous. Once data collection is complete, all data will be transferred to the researchers for analysis and long-term, secure storage and deleted from the iCare Adventure servers. The questionnaires have been made to be anonymous. Participants will not be identified by name or be identifiable by their responses.

Interview data will be transferred between the research team and the third party, transcription contractor via a secure, online portal or by courier. Transcripts will be de-identified. All data will be transferred to the researchers for analysis and long-term, secure storage and deleted by the third party contractor once transcription is complete. Master lists will be stored on a secure Canadian server only accessible to the research team.

## Discussion

Pediatric AGE is a common childhood illness representing a large burden on our healthcare system. Connecting parents and families to effective, evidence-based patient education is key to effective decision-making and therapeutic management of pediatric AGE. Digital KT tools offer a promising approach to communicate complex health information to parents and families. The evidence-based whiteboard animation video being evaluated in this pilot trial has been tailored to the needs of parents and families seeking care for pediatric AGE in EDs. This study will inform the design and conduct of a full-scale, effectiveness trial by gathering key data in four domains: (1) scientific, (2) process, (3) management, and (4) resource. These results will impact the emerging field of knowledge translation efforts targeting health consumers and advance the science on the best mode of patient education for acute childhood illnesses.

## Additional files

Additional file 1: Pre-intervention questionnaire. (DOCX 62 kb)

Additional file 2: Post-intervention questionnaire 1. (DOCX 61 kb)

Additional file 3: Post-intervention questionnaire 2. (DOCX 62 kb)

Additional file 4: Qualitative, semi-structured interview guide. (DOCX 13 kb)

## Abbreviations

AGE: Pediatric acute gastroenteritis
ED: Emergency department
KT: Knowledge translation
RCT: Randomized controlled trial
