# Supplementary material for: Evaluating a knowledge translation tool for parents about pediatric acute gastroenteritis: a pilot randomized trial
Source: Pilot Feasibility Stud. 2018 Aug 2;4:131. doi: 10.1186/s40814-018-0318-0 (PMC6090937; doi:10.1186/s40814-018-0318-0)
Supplement: Supplementary file 1 — Pre-intervention questionnaire. (DOCX 62 kb) [file 40814_2018_318_MOESM1_ESM.docx]

**Additional File 1: Pre-Intervention Questionnaire**

This first set of questions is about you. These questions are to help us understand the parents in our study as a group. Please answer all questions. Thank you.

1. What is your email address for a short, follow up questionnaire? [open text]
2. What is your gender?
   1. female
   2. male
   3. other [open text]
   4. prefer not to answer
3. What year were you born? [open text – limit to #s only]
4. What is your ethnicity?
   1. White
   2. Aboriginal (e.g., First Nations, Métis or Inuk)
   3. South Asian (e.g., East Indian, Pakistani, Sri Lankan, etc.)
   4. Chinese
   5. Black
   6. Filipino
   7. Latin American
   8. Arab
   9. Southeast Asian (e.g., Vietnamese, Cambodian, Laotian, Thai, etc.)
   10. West Asian (e.g., Iranian, Afghan, etc.)
   11. Korean
   12. Japanese
   13. Other [open text]
   14. prefer not to answer
5. Of what country are you a citizen?
   1. Canada by birth
   2. Canada by naturalization
   3. Other country [open text]
   4. prefer not to answer
6. What is your relationship status?
   1. single
   2. partnered
   3. other [open text]
   4. prefer not to answer
7. What is your highest level of education?
   1. high school diploma/equivalency
   2. certificate of apprenticeship/certificate of qualification as journeyperson
   3. college, CEGEP or other non-university certificate/diploma
   4. university certificate/diploma below bachelor level
   5. bachelor degree, university certificate/diploma above bachelor level
   6. graduate degree
   7. other [open text]
   8. prefer not to answer
8. How many children do you have [open text]
9. What is your relationship to the sick child?
   1. mother
   2. father
   3. other [open text]
   4. prefer not to answer
10. How old is the sick child? [open text]
11. What gender is the sick child?
    1. female
    2. male
    3. other [open text]
12. Has the sick child had vomiting (throw-up, puke) and diarrhea (poop) before?
    1. yes
    2. no
    3. unsure
13. When did the sick child’s vomiting (throw-up, puke) and diarrhea (poop) start?
    1. Today
    2. 1-2 days ago
    3. 3-5 days ago
    4. 6 or more days ago
    5. other [open text]
14. How many vomits (throw-up, puke) has the child had in the last 24 hours? [open text]
15. How many episodes of diarrhea (poop) has the child had in the last 24 hours? [open text]
16. Have other people in the house with the child had vomiting (throw-up, puke) and diarrhea (poop) in the last month?
    1. yes
    2. no
    3. unsure
17. Did you talk to or see another health professional before coming to the emergency department today? *(For example, did you call a doctor or nurse? Did you go to a walk-in clinic? Did you go to a pharmacy?)*
    1. Yes
       1. Who did you talk to/see? [open text]
    2. No
18. Did you look for information before coming to the emergency department today? *(For example did you call a family member or friend or did you look on the internet?)*
    1. yes
       1. Where did you look? [open text]
    2. no

Next, is a set of questions about childhood vomiting and diarrhea. Please answer all questions, if unsure about the answer please mark your best guess. Thank you.

1. Fill in the blank. Gastroenteritis is often caused by _________.

2. Choose the best option. Dehydration is when:

a. more fluids stay in the body than come out

b. more fluids come out of the body than stay in

c. you are thirsty

d. you have an upset stomach

e. none of the above

3. Check all that apply. A child is likely dehydrated if he/she:

□ has no tears when crying

□ has recently urinated

□ has cold hands and/or feet

□ has sunken eyes

□ asks for a drink

4. Check all that apply. You should take your child to the emergency department if he/she has vomiting and/or diarrhea and has:

□ been crying for more than 1 hour

□ not urinated (peed) in the last 12 hours

□ vomited (thrown-up) 2 times in the last 12 hours

□ multiple episodes of dark green vomit (throw-up, puke)

□ blood in diarrhea (poop)

5. Choose the best option. What types of fluids are encouraged to prevent/help dehydration?

a. no fluids

b. warm fluids

c. sugary fluids

d. clear fluids

e. any fluids the child will drink

6. Check all that apply. Which medications are helpful for a child with gastroenteritis?

□ medications for fever (like Tylenol)

□ medications for vomiting (like Gravol)

□ medications for diarrhea (like Imodium)

□ medications for upset stomach (like Pepto Bismol)

□ antibiotics

7. Fill in the blank. __________ is an example of a good oral rehydration solution to prevent and/or help dehydration.

8. True or False If child is not dehydrated, but is vomiting and/or having diarrhea over a few days, you should take him/her to see a doctor.

The last set of questions is about coming to the hospital emergency department today. Please think about your decision to bring your child to the hospital emergency department with vomiting and diarrhea. Please show how you feel about these statements by circling a number from 1 (strongly agree) to 5 (strongly disagree).


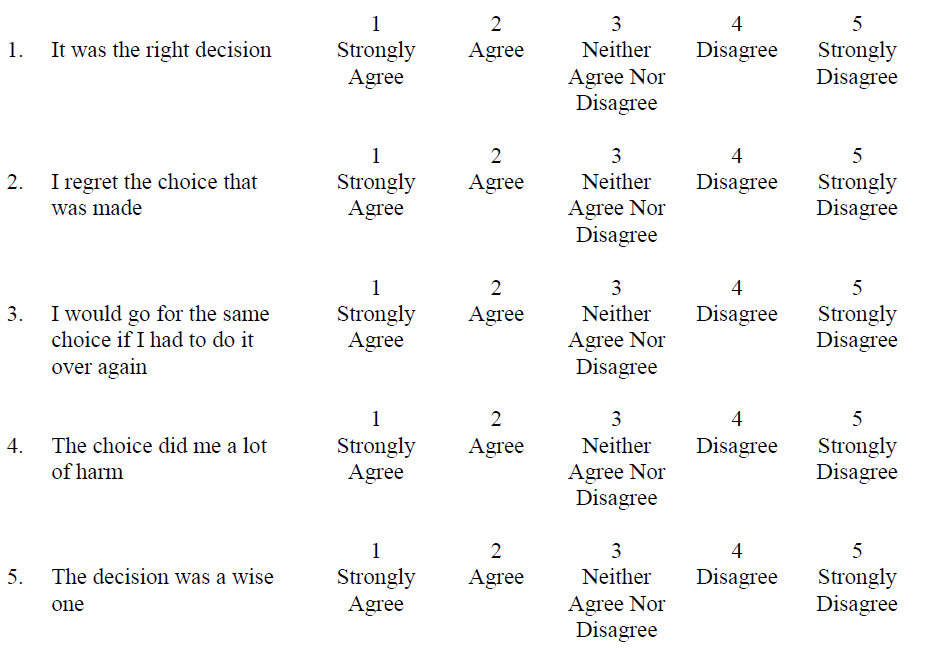


You will now watch a short, 3-minute video.
