# Supplementary material for: Evaluating a knowledge translation tool for parents about pediatric acute gastroenteritis: a pilot randomized trial
Source: Pilot Feasibility Stud. 2018 Aug 2;4:131. doi: 10.1186/s40814-018-0318-0 (PMC6090937; doi:10.1186/s40814-018-0318-0)
Supplement: Supplementary file 2 — Post-intervention questionnaire 1. (DOCX 61 kb) [file 40814_2018_318_MOESM2_ESM.docx]

**Additional File 2: Post-Intervention Questionnaire 1**

If you would like to view this video again when you leave the hospital emergency department, we can send you a link by email. Would you like to see the video again?

- 1. yes
  2. no

[This question is only for those in the experimental intervention group] Can we contact you for an in-person or telephone interview? The interview will be approximately 30 minutes to 1 hour long.

1. Yes
   1. Name [open text]
   2. Phone [open text]
   3. Best time to contact [open text]
2. No

Next, is a set of questions about childhood vomiting and diarrhea. Please answer all questions, if unsure about the answer please mark your best guess. Thank you.

1. Fill in the blank. Gastroenteritis is often caused by _________.

2. Choose the best option. Dehydration is when:

a. more fluids stay in the body than come out

b. more fluids come out of the body than stay in

c. you are thirsty

d. you have an upset stomach

e. none of the above

3. Check all that apply. A child is likely dehydrated if he/she:

□ has no tears when crying

□ has recently urinated

□ has cold hands and/or feet

□ has sunken eyes

□ asks for a drink

4. Check all that apply. You should take your child to the emergency department if he/she has vomiting and/or diarrhea and has:

□ been crying for more than 1 hour

□ not urinated (peed) in the last 12 hours

□ vomited (thrown-up) 2 times in the last 12 hours

□ multiple episodes of dark green vomit (throw-up, puke)

□ blood in diarrhea (poop)

5. Choose the best option. What types of fluids are encouraged to prevent/help dehydration?

a. no fluids

b. warm fluids

c. sugary fluids

d. clear fluids

e. any fluids the child will drink

6. Check all that apply. Which medications are helpful for a child with gastroenteritis?

□ medications for fever (like Tylenol)

□ medications for vomiting (like Gravol)

□ medications for diarrhea (like Imodium)

□ medications for upset stomach (like Pepto Bismol)

□ antibiotics

7. Fill in the blank. __________ is an example of a good oral rehydration solution to prevent and/or help dehydration.

8. True or False If child is not dehydrated, but is vomiting and/or having diarrhea over a few days, you should take him/her to see a doctor.

Compared to when you completed the first questionnaire, has your knowledge about childhood vomiting and diarrhea changed?

[insert 15 point scale]

-7: very great deal worse

-6: great deal worse

-5: good deal worse

-4: moderately worse

-3: somewhat worse

-2: a little worse

-1: almost hardly worse

0: the same

1: almost hardly better

2: a little better

3: somewhat better

4: moderately better

5: good deal better

6: great deal better

7: very great deal better

The next set of questions is about coming to the hospital emergency department today. Please think about your decision to bring your child to the hospital emergency department with vomiting and diarrhea. Please show how you feel about these statements by circling a number from 1 (strongly agree) to 5 (strongly disagree).


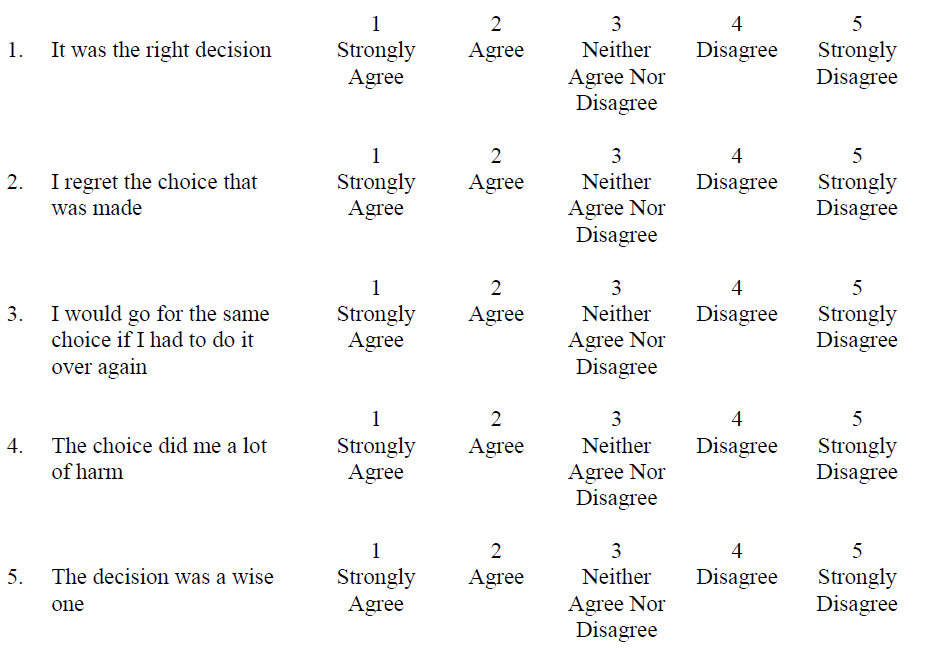


Compared to when you completed the first questionnaire, have your feelings about your decision to bring your child to the hospital emergency department with vomiting and diarrhea changed?

[insert 15 point scale]

-7: very great deal worse

-6: great deal worse

-5: good deal worse

-4: moderately worse

-3: somewhat worse

-2: a little worse

-1: almost hardly worse

0: the same

1: almost hardly better

2: a little better

3: somewhat better

4: moderately better

5: good deal better

6: great deal better

7: very great deal better

Thank you for your participation in our research project! Your responses are very valuable for our work. We will be sending you a final questionnaire by email to complete in 4 days. You will have 10 days to complete the questionnaire at your convenience. The questionnaire will take approximately 7-10 minutes to complete.
