# Supplementary material for: Evaluating a knowledge translation tool for parents about pediatric acute gastroenteritis: a pilot randomized trial
Source: Pilot Feasibility Stud. 2018 Aug 2;4:131. doi: 10.1186/s40814-018-0318-0 (PMC6090937; doi:10.1186/s40814-018-0318-0)
Supplement: Supplementary file 4 — Qualitative, semi-structured interview guide. (DOCX 13 kb) [file 40814_2018_318_MOESM4_ESM.docx]

**Additional File 4: Qualitative, Semi-Structured Interview Guide**

Thank you for taking the time to meet with me today. I would like to ask you several questions about your experience viewing a video. I am recording our conversation to ensure that we have an accurate summary of your opinions. All the information I collect will be kept confidential. You may refuse to answer any questions or leave the interview at any time. Do you have questions before we begin? Please feel free to ask questions at any time during the interview. Before we start, let’s watch the video again.

**play video**

1. What do you remember about watching this video in the hospital emergency department on [date]?
2. Tell me about that time your child was ill.
   1. Prompts: How did you know they were sick? What happened? What did you do?
3. Was the video helpful while you were in the hospital emergency department that day? How? Why/why not?
4. Tell me about your thought process as you were viewing the video.
   1. Prompt: Did you get the right information at the right time? Why/why not?
5. What did you do after you saw the video?
6. How did the video make you feel that day?
7. Do you feel that you learned something from the video? Why/why not?
8. Is there anything you would do differently next time your child has vomiting and diarrhea?
9. Thinking back, would it have been helpful to have access to the video after you left the hospital emergency department?
   1. Prompt: Would you have watched it again? When?
10. Is there anything you would change about your decision to go to the hospital emergency department on [date]?
    1. Prompt: Is there anything you know now that you wish you knew at the beginning?
11. Thinking back, when do you think would be the best time for you to have seen this video?
12. What did you think about using an online platform (program on the iPad and emailed link to survey) to participate in this research study?
    1. Prompt: Did you experience any issues with the technology? Was the iPad easy to use? Was it fun or interesting to complete a questionnaire like this while you were waiting in the hospital emergency department? Did having a link emailed to you make it easier to complete the follow-up questionnaire? Why/why not?
13. What did you think about the 3 questionnaires?
    1. Prompt: What did you think about the number of questions that were asked? What were your thoughts about the amount of time it took you to complete?
14. From your perspective, was there any difference between the questionnaires you completed in the hospital emergency department and the questionnaire you completed at home?
    1. Which was easier? Why?
15. Would you participate in future research studies similar to this one? Why/why not?
    1. Prompt: What did you like? What did you not like?

Thank you for participating in this interview.
